# Interstitial Lung Disease in Mixed Connective Tissue Disease: An Advanced Search

**DOI:** 10.7759/cureus.36204

**Published:** 2023-03-15

**Authors:** Juan Camilo Santacruz, Marta Juliana Mantilla, Gustavo Rodriguez-Salas, Igor Rueda, Sandra Pulido, Diana Cristina Varela, John Londono

**Affiliations:** 1 Spondyloarthropathies Research Group, Universidad de La Sabana, Chía, COL; 2 Rheumatology Department, Universidad Militar Nueva Granada, Bogotá, COL; 3 Rheumatology Department, Hospital General de Medellín, Medellín, COL

**Keywords:** treatment, progressive fibrosing phenotype, non-specific interstitial pneumonia, interstitial lung disease, mixed connective tissue disease

## Abstract

The spectrum of pulmonary manifestations associated with mixed connective tissue disease ranges from pulmonary hypertension and interstitial lung disease to pleural effusions, alveolar hemorrhage, and complications from the thromboembolic disease. Interstitial lung disease in mixed connective tissue disease is a frequently occurring entity, although in most cases it tends to be self-limited or slowly progressive. Despite this, a significant percentage of patients may present a progressive fibrosing phenotype, thus posing a great challenge regarding its therapeutic approach, given the scarcity of clinical studies that compare the efficacy of immunosuppressants available to date. Due to this, many recommendations are extrapolated from other diseases with similar characteristics such as systemic sclerosis and systemic lupus erythematosus. That is why it is proposed to carry out an advanced search of the literature in order to clarify its clinical, radiological, and therapeutic characteristics to achieve its evaluation from a holistic point of view.

## Introduction and background

Mixed connective tissue disease (MCTD) has been defined as an autoimmune disease characterized by high titers of anti-U1 ribonucleoprotein (U1-RNP) antibodies associated with other common characteristics described in other rheumatic diseases such as systemic sclerosis (SSc), polymyositis (PM), rheumatoid arthritis, and systemic lupus erythematosus (SLE), manifesting sequentially in time and being classified in principle as an undifferentiated connective tissue disease [1]. Pulmonary involvement in MCTD occurs frequently and is observed in about 75% of patients, with interstitial lung disease (ILD) and pulmonary hypertension (PH) being the most common forms of presentation [2]. MCTD associated with ILD accounts for approximately 6% of interstitial involvement of all connective tissue diseases, and about 24% show a progressive fibrosing phenotype [3]. The cohort studied by Reiseter has been one of the first to give a reliable estimate of the prevalence of ILD using high-resolution chest computed tomography (HRCT), being 40% [4]. Risk factors for the development of ILD include the presence of Raynaud's phenomenon, dysphagia, elevated C-reactive protein along with the presence of anti-Ro52 and anti-Sm antibodies [5]. Additionally, patients with digital ulcers are at greater risk of presenting severe and progressive ILD [6]. The non-specific interstitial pneumonia (NSIP) pattern is the most frequently described, with its distinctive characteristics being the presence of ground-glass opacities, reticular opacities, septal thickening, loss of volume, and predominantly peripheral or in the lower lobes [7]. ILD associated with MCTD is a little-recognized complication for which there are no clearly defined guidelines regarding its screening and therapeutic approach. Additionally, ILD, if not treated promptly, is usually progressive and severe in about 25% of patients after four years of follow-up [8]. That is why it is necessary to carry out an advanced search of the literature to consolidate a treatment guide and thus establish a comprehensive approach.

## Review

Methodology

**Figure 1:**
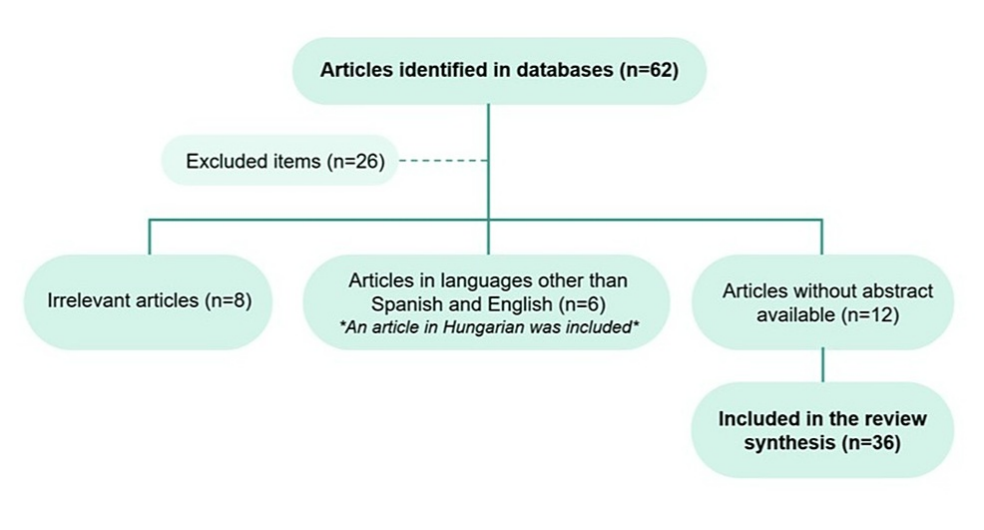
Search flow diagram

A non-systematic narrative review of the literature in English and Spanish was carried out (without identifying comparative studies to prepare a systemic review or meta-analysis). The review was carried out with the objective of having the most representative information available for the articles referenced in primary databases such as Pubmed, Embase, and Google Scholar. The MESH (medical subject headings) terms used were “Interstitial lung disease”, “Mixed connective tissue disease”, “Autoimmune diseases”, “Diagnosis”, and “Treatment”; and were combined using Boolean operators (AND, OR). Below is a flowchart detailing the search strategy (Figure 1).

Etiology and histopathology of ILD

Histopathological studies of the lung parenchyma performed in patients with MCTD have shown structural alterations similar to those of idiopathic pulmonary fibrosis. Although the pathogenic mechanism that triggers ILD is unknown, it may be similar to that of SLE and SSc [9]. Despite the fact that the pathogenic mechanism of ILD has similarities with SLE or SSc, the most studied theory is related to the higher prevalence of reflux, dilatation, and esophageal motility abnormalities shared by these diseases. One study observed statistically significant correlations between severe esophageal motor dysfunction and HRCT evidence of ILD along with a decrease in diffusing capacity of the lungs for the carbon monoxide (DLCO) test, predicting a reduction in total lung capacity [10]. To date, there are few pulmonary histopathological descriptions of patients with ILD associated with MCTD. One study succeeded in reviewing lung biopsies from 16 patients with MCTD, of whom the diagnosis of ILD could not be made using imaging techniques. All patients had findings compatible with NSIP, where 11 were classified as cellular NSIP with little or no fibrosis and the remaining five had significant fibrosis [11]. The histological changes described in lung specimens include the presence of alveolar septal infiltrates by lymphocytes, plasma cells, and type III collagen, these changes being indistinguishable from the ILD observed in patients with inflammatory myopathies [12]. Bronchoalveolar lavage generally shows a predominance of neutrophils, although in some cases eosinophils predominate and in others, a reduced CD4+/CD8+ ratio can be seen [13].

Clinical manifestations

The first symptoms that suggest lung involvement in MCTD are dry cough, dyspnea on exertion, and chest pain with pleuritic characteristics [14]. Because MCTD is a clinical combination of SLE, SSc, and PM, pulmonary manifestations of any of these disease processes can occur, although it should be investigated specifically for the other pulmonary manifestations typical of MCTD, such as pleural effusion, alveolar hemorrhage, PH, pulmonary vasculitis, infections, diaphragmatic dysfunction, obstructive airway disease, and thromboembolic disease [15].

Radiological features

**Figure 2:**
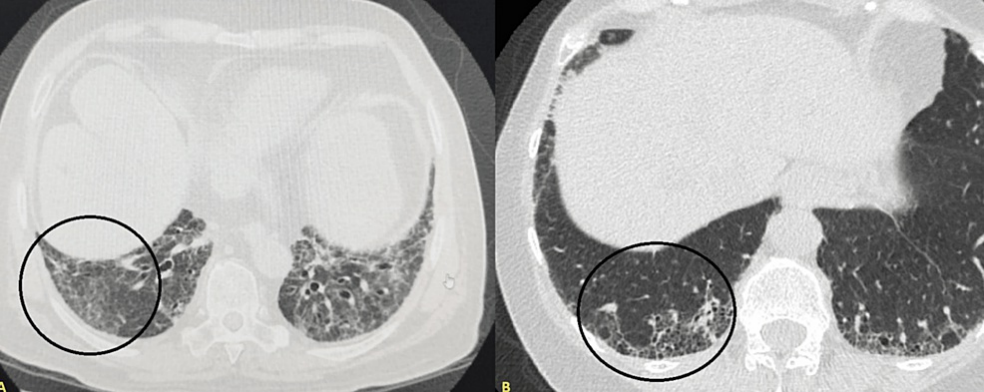
Most prevalent radiological patterns of ILD in MCTD Panel A (left): some features of NSIP on chest tomography are described; confluent ground-glass opacities along with reticular opacities and traction bronchiectasis. Panel B (right): some features of UIP on chest tomography are described; basal predominance, subpleural involvement associated with a reticular pattern, and honeycomb image. Other signs that occur more frequently in interstitial lung disease associated with connective tissue diseases may also be seen, such as the straight edge sign (isolation of fibrosis at the lung bases without substantial extension to the lateral margins of the lungs on coronal slices), exuberant honeycomb sign (extensive cyst formation within the lungs involving more than 70% of the fibrotic portions), and the anterior upper lobe sign (concentration of fibrosis within the anterior aspect of the upper lobes and concomitant lower lobe involvement). Reference: [22] ILD: interstitial lung disease; MCTD: mixed connective tissue disease; NSIP: non-specific interstitial pneumonia; UIP: interstitial pneumonia

The characteristics of ILD in MCTD are similar to those observed in patients with SLE, SSc, and PM. HRCT findings are heterogeneous, including the spectrum of pneumonitis, fibrotic NSIP, and pericardial or pleural effusion [16]. Although the course of pulmonary fibrosis is usually slow-progressing, as in SSc, it is associated with a higher risk of mortality [17]. The usual pattern of interstitial pneumonia (UIP) has also been reported; in fact, in a series of cases, it is described that most of the patients debuted with evidence of interstitial pneumonitis associated with bronchiectasis, having a subsequent transition to a pattern defined as NSIP or UIP [18]. Centrilobular nodules, honeycombs, and consolidations are uncommon findings. Cases of alveolar hemorrhage and organizing pneumonia have been described more in relation to acute exacerbations [19,20]. Severe fibrosis has been associated with advancing age, but not with the duration of diagnosis or with smoking. It seems that the presence of arthritis protects against the progression of lung disease, possibly indicating that patients with MCTD with characteristics similar to those of SLE are less likely to develop it [21]. Two examples of ILD in patients with MCTD are presented below, where the most representative characteristics of each radiological pattern are described (Figure 2).

Prognosis and clinical follow-up

It has been shown that patients with MCTD and ILD have significantly lower survival at five and 10 years, mainly in those who compromise more than 10% of the total lung volume by HRCT [23]. There are currently no established guidelines regarding the screening and treatment of ILD in MCTD, although it has increasingly been shown to be an underdiagnosed condition. According to the available evidence, follow-up is carried out according to the ILD recommendations in SSc at intervals of three to six months with pulmonary function tests [24]. Patients with more extensive fibrosis have marked decreases in DLCO and forced vital capacity (FVC), suggesting restrictive lung disease on spirometry along with decreased six-minute walking distance [25]. Despite the fact that today there are better treatment opportunities for extensive interstitial lung involvement, most patients with SSc, inflammatory myopathies, and MCTD coincide with a median survival of fewer than five years [26].

Treatment

Distinguishing MCTD from other rheumatic diseases, including overlap syndrome, is challenging because about 61% are misdiagnosed at the time of initial classification [27]. Additionally, some patients subsequently differentiate to another systemic disease over time, although true phenotypic conversion is rare [28]. On the other hand, about 47% of the patients with MCTD meet the classification criteria for SLE, so there may be a substantial variation regarding the treatment of interstitial involvement of both entities [29]. In order to define whether the patient requires immunosuppressive treatment, the severity and rate of progression must be evaluated together with the indications for treatment of the extrapulmonary manifestations of MCTD (pericarditis, pleurisy, myositis, and myocarditis). Treatment is generally reserved for patients who have a fibrosing radiological pattern with evidence of progression demonstrated by a fall in FVC or DLCO of at least 10%, a decrease in relative FVC of 5 to 10%, an increase in the frequency of respiratory symptoms and a greater extension of interstitial involvement in the chest HRCT [30].

Current Evidence of Available Immunosuppressants

According to the guidelines for the treatment of ILD in SSc, immunosuppressive therapy initially with mycophenolate mofetil (MMF) is preferred instead of cyclophosphamide as it presents fewer adverse effects and therefore better tolerance [31]. Tocilizumab is FDA-approved for the treatment of ILD to decrease the rate of progression of interstitial involvement in patients with early-stage diffuse SSc who are intolerant of MMF. Despite this, it has not been shown that there are elevated levels of IL-6 in patients with ILD associated with MCTD or that it intervenes in the progression of fibrosis, so it is not considered in this context as an alternative therapy [32]. The RECITAL study was a multicenter, prospective, randomized, double-blind, phase 2b study, which included 145 patients, randomized 1:1 to each treatment arm, comparing rituximab at a dose of 1 g administered intravenously, twice with an interval of two weeks, with intravenous cyclophosphamide administered monthly at a dose of 600 mg/m^2^ in patients with ILD secondary to inflammatory myopathies (including anti-synthetase syndrome), SSc, and MCTD, having follow-up for 48 weeks from the first dose. Although rituximab did not demonstrate superiority in the primary outcome (change in FVC from baseline to week 24), it was associated with fewer adverse events and lower glucocorticoid exposure during the 48-week follow-up. It is relevant to take into account that only 16 MCTD patients were included in the study and a larger sample is required to be able to generalize the results, however, to date, this is the only study that has included MCTD patients with ILD with the objective of evaluating therapeutic response [33].

Current Evidence for Antifibrotics and Glucocorticoids

The INBUILD study evaluated the efficacy of nintedanib, a tyrosine kinase inhibitor, including in the subgroup analysis 170 patients with ILD related to autoimmune diseases, demonstrating a rate of decline in FVC over 52 weeks of -75.9ml/year in the nintedanib group versus -178ml/year with placebo, with diarrhea and nausea being the most frequent adverse effects [34]. The dose of nintedanib is 150 mg orally every 12 hours with an indication to perform liver function tests before starting it since it is contraindicated in patients with Child-Pugh B or C liver failure. Nintedanib can cause fetal harm so a negative pregnancy test is imperative in women of childbearing potential before starting it, also guaranteeing an effective contraceptive method during the time of treatment [35]. There are real-life studies that support that nintedanib has been associated with an attenuation of the decline in lung function regardless of the severity of the ILD, so today it is an additional treatment strategy [36]. The dose of glucocorticoids is not yet clearly established in this context, although it seems reasonable to define a dose of no more than 10 mg per day of prednisolone for patients in whom the characteristics of SSc predominate while those who meet characteristics of PM or SLE would indicate a dose of 1 mg/kg per day for four weeks to then gradually withdraw [37].

**Figure 3:**
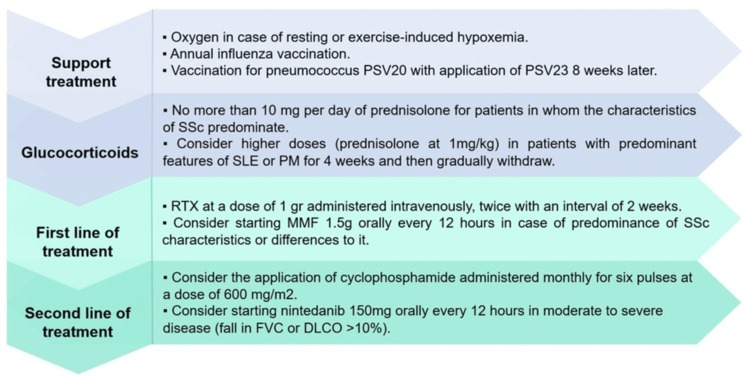
Suggested therapeutic algorithm for patients with ILD associated with MCTD DLCO: diffusing capacity for carbon monoxide; FVC: forced vital capacity; MMF: mycophenolate mofetil; PM: polymyositis; RTX: rituximab; SLE; systemic lupus erythematosus; SSc: systemic sclerosis; ILD: interstitial lung disease; MCTD: mixed connective tissue disease References: [31,33,34]

Figure 3 describes the therapeutic approach for patients with ILD in MCTD according to the available levels of evidence and Table 1 shows some aspects to consider in special situations and the duration of each treatment.

**Table 1:** Aspects to consider regarding mycophenolate mofetil and rituximab, drug interactions, and treatment time MMF: mycophenolate mofetil; PPIs: proton pump inhibitors

Drug	Mycophenolate mofetil	Rituximab	Reference
Aspects to consider	The maximum dose should be reduced in patients with end-stage renal disease (maximum of 2g per day when the glomerular filtration rate is less than 25 ml/minute)	Patients should receive the appropriate vaccinations, ideally four weeks prior to administration	[38]
Drug interactions	Dosing of antacids, PPIs, and mineral supplements should be separated by at least two hours (alter serum MMF concentrations)	To prevent infusion reactions, premedication with chlorpheniramine, acetaminophen, and methylprednisolone is indicated 30 minutes before each infusion	[39]
Treatment time	24 months, although it is preferred to continue for several years	Extended treatment time, if there is clinical stability for two years, the dosing interval could be spaced	[40]

## Conclusions

Although the main causes of death in relation to MCTD are related to cardiovascular complications derived from PH, ILD is increasingly recognized as an important cause of morbidity and mortality. Despite the fact that MCTD has a lower prevalence compared to other autoimmune diseases such as SLE, SSc, or inflammatory myopathies, the diagnostic and therapeutic approach to interstitial involvement continues to be a great challenge given the scant evidence available to date. It is important to emphasize the need to include only patients with MCTD with interstitial involvement in clinical trials so as not to extrapolate treatments from other related autoimmune diseases and to standardize the dose of glucocorticoids for each context. To date, mycophenolate or rituximab has been considered as induction therapy associated with variable doses of glucocorticoids depending on their predominant phenotype.
